# Peritumoral Brain Edema in Relation to Tumor Size Is a Variable That Influences the Risk of Recurrence in Intracranial Meningiomas

**DOI:** 10.3390/tomography8040166

**Published:** 2022-08-08

**Authors:** Alessandro Frati, Daniele Armocida, Umberto Aldo Arcidiacono, Alessandro Pesce, Giancarlo D’Andrea, Fabio Cofano, Diego Garbossa, Antonio Santoro

**Affiliations:** 1 IRCCS “Neuromed” Pozzilli, 86170 Isernia, Italy; 2 Human Neurosciences Department, Neurosurgery Division, “Sapienza” University, 00135 Rome, Italy; 3 Neurosurgery Division, Santa Maria Goretti Hospital, Via Guido Reni, 04100 Latina, Italy; 4 Neurosurgery Department of Fabrizio Spaziani Hospital, 03100 Frosinone, Italy; 5 Unit of Neurosurgery, AOU Città della Salute e della Scienza, 10126 Torino, Italy; 6 Neurosurgery Unit, Department of Neuroscience “Rita Levi Montalcini”, University of Turin, 10124 Turin, Italy

**Keywords:** meningioma, neurosurgery, peritumoral brain edema, tumor recurrence

## Abstract

Peritumoral brain edema (PBE) is common in intracranial meningiomas (IM) and can increase their morbidity. It is not uncommon for a neurosurgeon to confront meningiomas with a large proportion of PBE independently from the site and size of the contrast-enhancing lesion with increased surgical risks. We performed a retrospective review of 216 surgically-treated patients suffering from IM. We recorded clinical, biological, and radiological data based on the rate of tumor and edema volume and divided the patients into a group with high Edema/Tumor ratio and a group with a low ratio. We investigated how the ratio of edema/lesion may affect the outcome. Multivariate analysis was performed for the two groups. Smokers were found to be more likely to belong to the high-rate group. The edema/tumor ratio did not affect the surgical radicality; however, independently of the biological sub-type, WHO grading, and EOR, a higher frequency of recurrence is shown in patients with a high edema/tumor ratio (70.5% vs. 8.4%. *p* < 0.01). There is evidence to suggest that the blood-brain barrier (BBB) damage from smoke could play a role in an increased volume of PBE. The present study demonstrates that IMs showing a high PBE ratio to tumor volume at diagnosis are associated with a smoking habit and a higher incidence of recurrence independently of their biological type and grading.

## 1. Introduction

**Figure 1:**
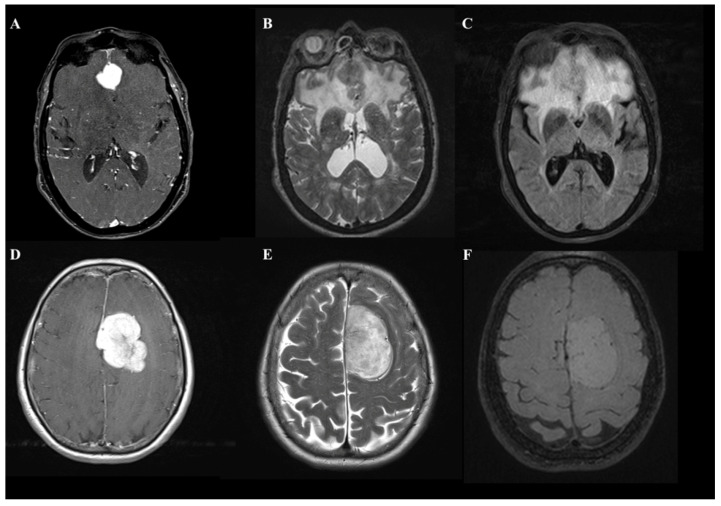
A comparison of two cases of patients with frontal lobe meningioma with an asymptomatic onset that, on MRI images, document entirely different volumes of edema. In the first case, T1-weighted with MDC (**A**), T2-weighted (**B**), and FLAIR (**C**) images show a 2 cm para-alpine lesion with edema involving the entire frontal lobe. In the second case, the identical sequences (**D**–**F**) show a more than 4.5 cm lesion in diameter with a volume of edema almost absent.

Meningiomas are benign neoplasms arising from meningoendothelial cells [1]. They are the most frequent intracranial tumor in the adult population. Although intracranial meningiomas (IM) are typical extra-axial tumors, the occurrence of peritumoral brain edemas (PBE) are not rare, affecting between 38% to 67% of IM patients [1,2,3]. It is well recognized that a large proportion of PBE can increase morbidity and mortality [2] and determine brain displacement, increase intracranial pressure [3], and add the risk of perioperative seizures [4]. In daily clinical practice, it is not uncommon for a neurosurgeon to confront the diagnosis of an IM with a large proportion of edema irrespective of the site and size of the contrast-enhancing lesion (Figure 1).

Despite the relatively large number of studies on this topic [1,5,6,7], the mechanisms by which an IM produces PBE are not fully clarified. Furthermore, none of the published hypotheses on molecular mechanisms can explain the genesis of PBE and its role in clinical outcomes [3]. In this retrospective study, we investigated several possible clinical and biological variables that may result in a higher proportion of edema at IM diagnosis. We subsequently investigated how a high edema/lesion ratio may affect clinical and surgical outcomes in a homogeneous cohort of patients suffering from IMs.

## 2. Materials and Methods

We performed an Institutional retrospective review of the imaging of a consecutive series of surgically-treated patients who were suffering from IMs that were histologically confirmed by the World Health Organization (WHO) in 2021 and operated on in Sapienza Neurosurgery Department of Rome (Italy) and the Neurosurgery department of Hospital Spaziani of Frosinone (Italy) between January 2016 and December 2020. We collected a total of 216 patients affected by IMs.

For all included patients, we recorded sex, age, length of hospitalization and follow-up, clinical onset, smoke habit, comorbidities, and performance status (evaluated using the Karnofsky performance scale, KPS) at the moment of diagnosis. The neurological and clinical examinations, as well as the presence of seizure at onset, were recorded as well.

On the ground of the final histological diagnoses, we recorded WHO grading with subtypes, immunohistochemistry with Ki67, and Progesterone Receptor (PR) expression. Concerning the radiological evaluation, we recorded parameters such as the location of the lesion, the involvement of the subtentorial compartment, large tumor diameter (measured in cm), and tumor volumes (measured in cm^3^) using isotropic volumetric T1-weighted sequences before and after intravenous administration of the paramagnetic contrast agent (gadolinium). We used T2-weighted and Fluid Attenuated Inversion Recovery (FLAIR) sequences to obtain the edema volumes (measured in cm^3^ before antiedemigen therapy). The volume of the contrast-enhancing lesion and edema was calculated by drawing a region of interest (ROI) in a Volumetric enhancing post-contrast study weighted in T1 (a multivoxel study) and T2, conforming to the margins of the contrast-enhancing lesion with the software Horos [8] following our previously published institutional protocol for IM [9].

Starting from these radiologic parameters, we calculated the ratio between tumor volume and edema volume and obtained a dichotomous variable dividing the entire cohort into two subgroups:

**Group A**, High-ratio Edema/Tumor: the volume of edema is equal to or greater than that of the contrast-enhancing lesion (with a numerical rate < 1);

**Group B**, Low-ratio Edema/Tumor: the volume of edema is lesser than that of the contrast-enhancing lesion (with a numerical rate > 1) or lesions without quantifiable edema.

Overall survival (OS) was recorded in months and measured from the date of diagnosis to the date of death or the date of last contact, if the patient was still alive. Clinical information was obtained by our institution’s digital database, whereas telephone interviews were used to obtain OS data. We recorded the status of performance (using KPS) for each patient after the surgical procedure at one month, six months, and at the last clinical evaluation. A particular focus was on the KPS results; this parameter was considered, as previously observed, and critically associated with a better result in terms of quality of life. We evaluated the presence of complications, recurrence, and the consequent need for further treatments. We investigated whether the large diameter on radiological diagnosis indicates different OS, grading, immunohistochemical characteristics, and clinical/neurological outcome.

### Statistical Methods

The sample was analyzed with SPSS version 18. We examined the relationship between these factors and brain edema through univariate and multivariate analyses. Comparisons between nominal variables were made with the Chi-squared test. The extent of resection (EOR, measured with Simpson Grade) means was compared with One Way and Multivariate ANOVA analysis, Contrast analysis, and Post-Hoc Tests. Continuous variable correlations have been investigated with Pearson’s Bivariate correlation. The threshold of statistical significance was considered *p* < 0.05.

## 3. Results

All relevant details on patient demographics are summarized in Table 1.

**Figure 2:**
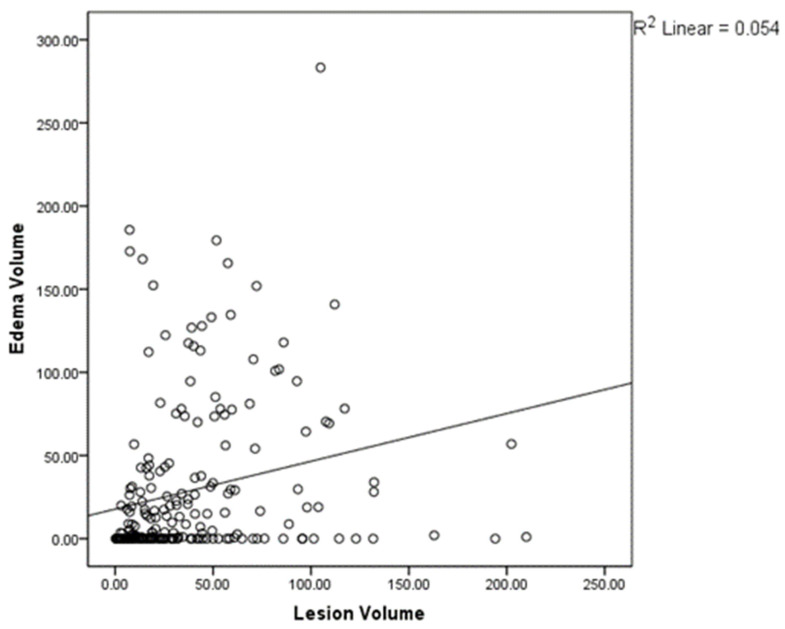
Pearson’s bivariate correlation plot demonstrating the presence of a positive correlation between the contrast-enhancing lesion volume and the edema volume as measured in T2-FLAIR (*p* < 0.01).

The radiological analysis confirms the presence of a positive correlation between the increasing contrast-enhancing lesion volume and the edema volume measured in T2-FLAIR by Pearson’s bivariate correlation analysis (*p* < 0.01, Figure 2). The relevance of the division in two groups is confirmed by the fact that, while there is a significant difference between the average volumes of edema between group A and group B (*p* < 0.01), this difference is lost when comparing the average volumes of contrast-enhancing lesions (*p* = 0.99).

**Figure 3:**
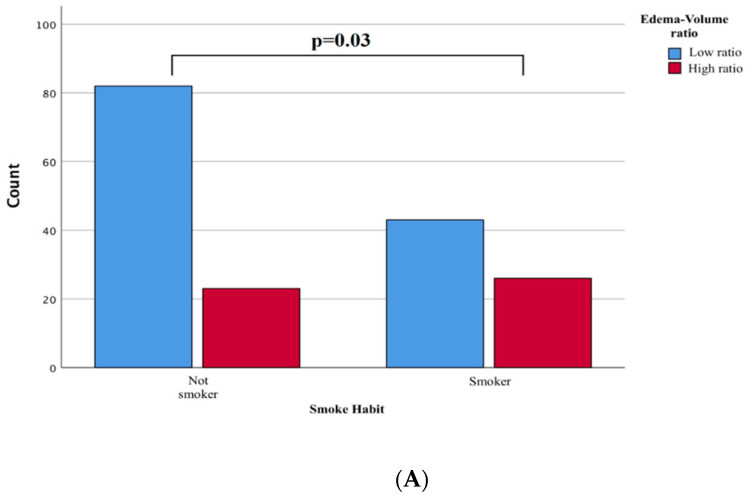

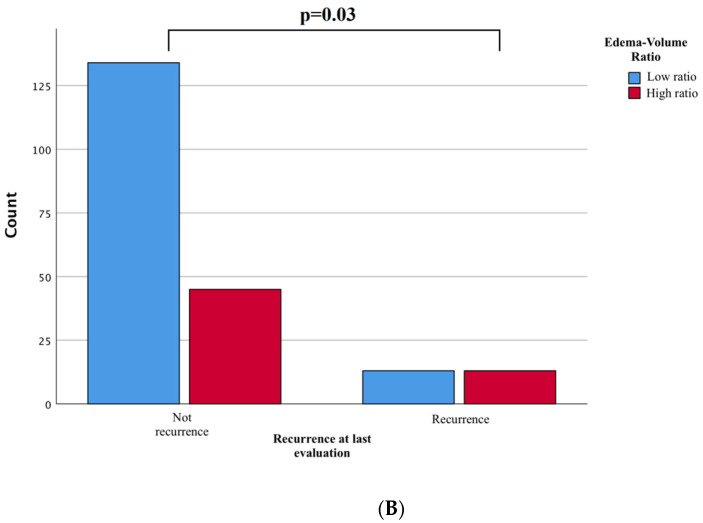
(**A**): Chi-square Comparison Analysis shows statistically significant differences in the smoking habit in subjects with a high edema-to-tumor volume ratio (*p* = 0.03). (**B**): significantly increased risk of developing recurrence independently compared with meningiomas with a low ratio (*p* = 0.03).

From a clinical perspective, the comparison between the two subgroups showed no significant differences in variables such as age (*p* = 0.15), sex (*p* = 1), and comorbidities (*p* = 0.32). Conversely, a history of smoking intake was more frequently associated with the group with a high rate of edema/tumor (*p* = 0.03, Figure 3A). The pathogenesis could be linked to a previously reported blood-brain barrier (see discussion below), although the written result should be interpreted cautiously as a statistical association.

Clinical onset in subjects with a high edema/tumor ratio is more frequently associated with the presence of seizures (*p* = 0.05) and with an insignificant difference in performance (measured by KPS) at onset (mean 70 vs. 80, *p* = 0.18).

Localization does not affect the edema/tumor ratio (ANOVA *p* = 0.34), although convexity meningiomas were statistically associated with a higher edema/tumor ratio than deep or subtentorial meningiomas (*p* = 0.06). Surprisingly, we found a statistically significant higher incidence of recurrences among the high ratio tumors (*p* = 0.03, Figure 3B).

Biological variables such as Ki67 percentage, biological subtype of the lesion, and progesterone expression do not significantly correlate with the total edema volume or the value of the edema to lesion ratio. It is confirmed that the grading of meningiomas is an independent variable of outcome, but it has no direct correlation with edema volume.

Regarding the outcome variables, we compared the two groups with multivariate analysis by considering WHO type, biological type, location, and EOR. The analyses ruled out a higher rate of complications (including ischemia *p* = 0.39 and infection *p* = 1), postoperative seizures (*p* = 0.49), mortality, and hospitalization time (*p* = 0.51) between the two groups (*p* = 1). Surgical radicality, as measured by Simpson’s grade, was not influenced by the edema/tumor ratio (*p* = 0.42); however, a higher frequency of recurrence is shown in patients with high edema/tumor ratio (70.5% vs. 8.4%. *p* < 0.01) independent of the biological subtype, WHO grading, and EOR.

**Figure 4:**
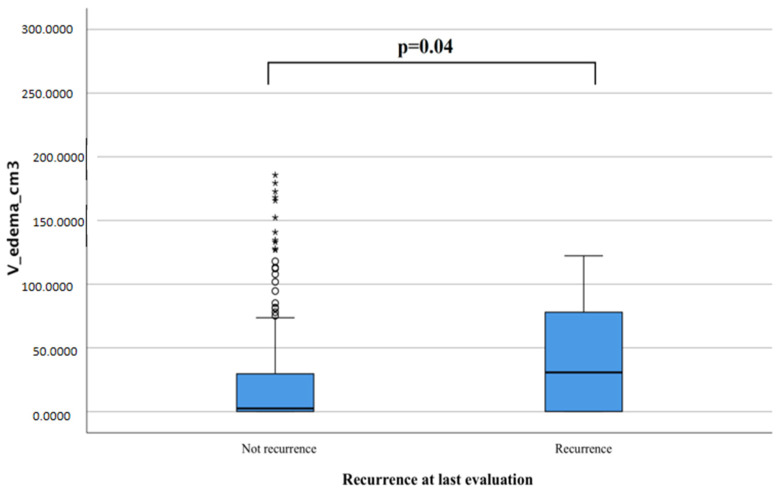
In the box letter graph, there is a significant difference between the group of patients developing recurrence with the total volume of edema (*p* = 0.04). This difference appears to be independent of WHO grade.

We obtained similar data by measuring the relationship between edema volume and risk of recurrence with t-student tests and found that IMs that presented more significant tumor edema at diagnosis were more likely to recur if compared to those that never recurred (42.3 cm^3^ vs. 26.62 cm^3^, *p* = 0.04, Figure 4).

## 4. Discussion

PBE is found in approximately 50% of meningiomas [1], and it may be present to varying degrees in an unpredictable fashion [9]. Although meningiomas are indolent, slowly growing benign tumors, they are often accompanied by a brain edema that causes clinical symptoms [6,7,10]. From a clinical perspective, a conspicuous PBE could negatively affect perioperative morbidity and mortality in meningioma surgery [11]; hence, significant brain edemas may be preoperatively associated with severe neurologic deficits, which intraoperatively limits the surgical field exposure and increases the risk of unsatisfactory outcomes in the postoperative period [12].

Further, the loss of BBB integrity and selective permeability, impact on BBB transport mechanisms, post-traumatic cerebral edema formation, and significant pathophysiological factors may exacerbate BBB dysfunctions in trauma [12,13,14,15,16] and tumors [16].

Several mechanisms have been hypothesized in the literature as to why BBB damage may be related to increased development of cerebral edema; among the various hypotheses suggested is the possible role of cigarette smoking. Abundant evidence indicates the central role of oxidative stress in endothelial dysfunction and subsequent BBB damage at the cerebrovascular level. Exposure to tobacco smoke, even at nontoxic concentrations, induces a robust inflammatory response in cells affecting the cerebrovascular endothelium and circulating immune cells. The inflammatory response and reduced cerebral blood flow, common in chronic smokers, result in excessive damage to the BBB, which suggests its contribution to PBE [16,17].

In the present investigation, we focus on the relationship between tumor size and the volume of edema it generates rather than on the absolute value of the volume of PBE. However, there is still no solid explanation for why some lesions demonstrate a high edema/tumor ratio. We identified a subgroup of IMs with a notably high tendency to generate PBE. We found that high ratio edema/tumor IM patients more frequently debut with seizures.

The possible role of smoking in the development of meningioma is still unclear. Although the nicotine itself does not affect the blood-brain barrier (BBB), it seems that there is an increase of permeability with the loss of function in vivo in the smokers of this section [13,14]. PBE originates in the margin regions of the tumor, suggesting a vasogenic origin and a pathogenic mechanism that primarily links IMs with BBB disorders; alternatively, the disruption of BBB is supposedly linked to the development of brain edemas in different conditions [15,16]

From a biological point of view, no data was found to be associated with the development of IM with a high proportion of PBE [18]. Although previous studies reported that angiomatous and meningothelial types tend to be associated with more significant brain edemas [19], it was not possible to confirm such a clear association in our cohort.

A study from Bečulić H et al. reported an association between the percentage of ki67 expression and the presence of PBE in disagreement with our results. However, that study first correlated the expression of ki67 values with arachnoid invasion capacity, size, and grading; our study only partially confirms these data, which merits further investigation by analyzing the various histological subtypes in a more extensive case series in future work [20].

We confirmed that WHO high-grade meningiomas independently correlate with a worse outcome [21] and an elevated risk of recurrence in patients. However, the most interesting finding of our study demonstrates that, although there is no direct relationship between tumor grading and the number of edemas, more edematous meningiomas have a higher risk of recurrence over time independent of the Simpson grade achieved with surgery.

We propose three possible explanations for this finding. Firstly, an increased BBB permeability could be associated with an increased vascular supply to the surgical cavity [19,22] during the post-surgical phase and may increase the risk of recurrence or regrowth in predisposed subjects. The presence of edemas suggests a greater predisposition to recur independently of the patient’s grading, Ki-67 expression, and hormonal receptor status. Secondly, the swollen brain parenchyma is typically fragile and difficult to manipulate safely, which could hide microscopic “islands” of the remnant and eventually produce a regrowth; the differences in vascularity among IMs is another possible explanation that may account for such a large subset of meningiomas being edemigenous independent of the tumor volume and biological status. Smith et al. [23] found that tumors with increased cellularity, vascularity, and mitotic activity had edemas more frequently. Challa et al. [24] reported that more vascular tumors tend to break capillary endothelial tight junctions, leading to increased permeability to water. High vascularity may also cause increased water content in the cancer.

In our results and experience, the cause of PBE associated with meningioma is most likely multifactorial. The location of the dural attachment can be considered a factor that is predisposed to developing more significant edemas. This is also demonstrated in our study, where anterior cranial fossa meningiomas compared to other locations and convexity meningiomas compared to deep meningiomas are associated with more significant peritumoral edema. However, this finding must be, in our opinion, considered with caution; anterior convexity meningiomas are the most common and most likely ones to reach large sizes before becoming symptomatic, so there may be selection bias.

The two groups examined do not present significant differences in clinical outcomes, EOR, and postoperative complications once surgically treated. Performance status as measured with the KPS does not demonstrate differences between the highly edematous tumors compared to their standard counterparts. However, we suggest a more careful and restricted clinical and radiological follow-up for this particular subgroup of patients.

### Limitations

A potential source of bias is expected to derive from the exiguity of the sample, which, considering the selected endpoints, presents an excellent post-hoc statistical estimated power (difference between two independent means; 1 − β = 0.9488 for α 0.05 and effect size 0.5) and thus provides highly reliable conclusions.

## 5. Conclusions

Many investigations have been carried out to determine the pathogenesis of PBE, but the exact pathogenesis in meningiomas is still unclear and debated, although it is most likely multifactorial.

The present study reports an extensive collection of intracranial meningiomas in which there is a wide variability of PBE to tumor volume; it appears that the high ratio between these two parameters correlates with the presence of smoke habit at diagnosis, suggesting that the blood-brain barrier (BBB) damage from smoke could play a role in an increased volume of PBE. It also shows that IMs with a high volume of PBE are correlated with a higher incidence of recurrence regardless of the biological type and histologic grading.

## Figures and Tables

**Table 1:** Analysis of groups.

216 Patients	Group A High Rate (61 pts)	Group B Low-Rate (155 pts)	*p*-Value
**Female sex**	41	113	0.41
**Age (mean)**	60 ± 14.91	59.7 ± 13.6	1
**Follow-up (months)**	45.93	46.2	1
**Hospitalization (days)**	17.77	19.82	0.77
**Smoke**	26	43	**0.02**
**Comorbidity**	18	50	0.32
**High grade WHO**	12	25	0.55
**Convexity Location**	26	66	0.06
**Subtentorial**	1	17	0.27
**Supratentorial**	60	127	0.27
**Frontal Location**	34	61	0.07
**Diameter (mean cm)**	4.6	4.48	1
**Tumor volume (mean cm^3^)**	35.42	37.87	0.99
**Edema Volume (mean cm^3^)**	80.68	7.72	**<0.01**
**ki67%**	8%	5%	0.26
**PR+**	8	20	0.55
**Clinical debut**	Incidental Dizziness Focal deficit Headache Seizure Mental alteration	Incidental Dizziness Focal deficit Headache Seizure Mental alteration	1
**Seizure at debut**	19	30	**0.05**
**KPS at onset (mean)**	70	80	
**Post-operative KPS**	70	70	
**KPS at last evaluation**	80	90	
**Seizure after treatment**	9	20	0.49
**Complications**	14	37	0.51
**Recurrence (no. patients)**	43–70.5%	13–8.4%	**0.01**

Bold in *p*-Value: *p*-Value < 0.05.

## Data Availability

Available on request daniele.armocida@uniroma1.it (accessed on 1 September 2021).
